# Ptk7 and Mcc, Unfancied Components in Non-Canonical Wnt Signaling and Cancer

**DOI:** 10.3390/cancers8070068

**Published:** 2016-07-16

**Authors:** Norris Ray Dunn, Nicholas S. Tolwinski

**Affiliations:** 1 Agency for Science Technology and Research (A*STAR) Institute of Medical Biology, 8A Biomedical Grove, #06-06 Immunos, Singapore 138648, Singapore; ray.dunn@imb.a-star.edu.sg; 2 Division of Science, Yale-NUS College, Singapore 138610, Singapore; 3 Department of Biological Sciences, Centre for Translational Medicine, NUS Yong Loo Lin School of Medicine, National University of Singapore, 14 Medical Drive, Level 10 South, 10-02M, Singapore 117599, Singapore

**Keywords:** Wnt, Ptk7, Mcc, PCP, cancer

## Abstract

Human development uses a remarkably small number of signal transduction pathways to organize vastly complicated tissues. These pathways are commonly associated with disease in adults if activated inappropriately. One such signaling pathway, Wnt, solves the too few pathways conundrum by having many alternate pathways within the Wnt network*.* The main or “canonical” Wnt pathway has been studied in great detail, and among its numerous downstream components, several have been identified as drug targets that have led to cancer treatments currently in clinical trials. In contrast, the non-canonical Wnt pathways are less well characterized, and few if any possible drug targets exist to tackle cancers caused by dysregulation of these Wnt offshoots. In this review, we focus on two molecules—Protein Tyrosine Kinase 7 (Ptk7) and Mutated in Colorectal Cancer (Mcc)—that do not fit perfectly into the non-canonical pathways described to date and whose roles in cancer are ill defined. We will summarize work from our laboratories as well as many others revealing unexpected links between these two proteins and Wnt signaling both in cancer progression and during vertebrate and invertebrate embryonic development. We propose that future studies focused on delineating the signaling machinery downstream of Ptk7 and Mcc will provide new, hitherto unanticipated drug targets to combat cancer metastasis.

## 1. Introduction

Human cancer is often caused by mutations in genes that regulate embryonic development and adult homeostasis. Many of these genes are part of an intricate network of signals controlling cellular behavior throughout the complex process of embryogenesis. One such signal, involved in a variety of contexts, is known as the Wnt signaling pathway [1,2,3,4,5]. Wnt ligands transmit signals from cell to cell across membranes to regulate gene expression and protein localization [6,7]. Too much, or too little, Wnt leads to developmental defects and/or disease. Many aspects of Wnt polarity pathways and their function in generating cellular asymmetry are understood, but the basic cell biology of how these secreted ligands influence cell shape and cell movement remains unclear [8,9,10]. Most importantly, the combination of cell movement and growth is the basis of metastasis and tumor invasiveness [11].

**Figure 1:**
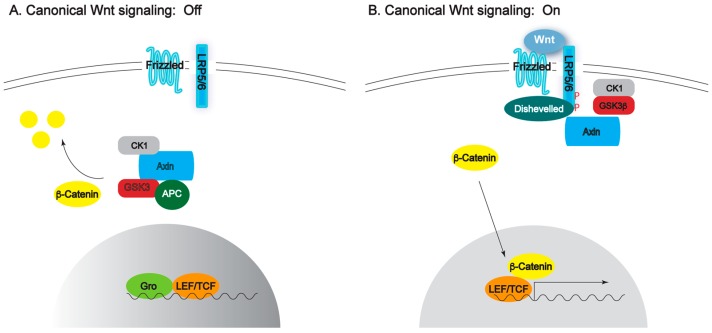

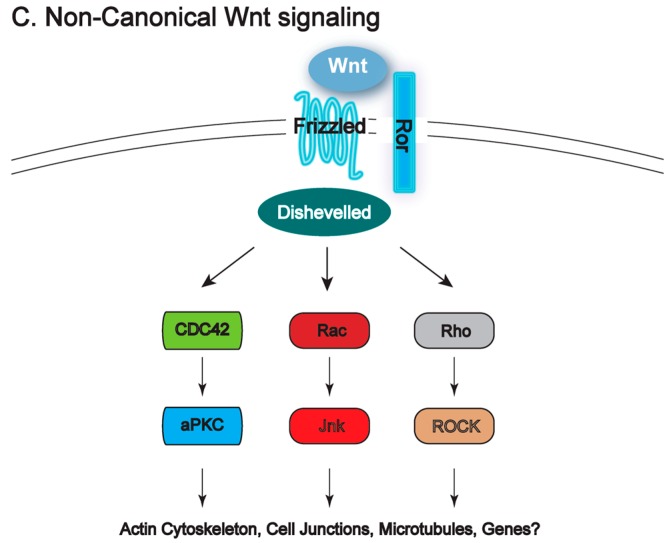
A schematic representation of the Wnt pathway in off (**A**) and on (**B**) conformations and the non-canonical Wnt pathway (**C**). (**A**) In the absence of Wnt ligand, the destruction complex forms in the cytoplasm bringing together APC and Axin, and presenting β-catenin for phosphorylation by CK1 and GSK3. Phosphorylated β-catenin is ubiquitylated and degraded by the proteasome, and does not enter the nucleus where gene expression is repressed by TCF in complex with the repressor Groucho/TLE; (**B**) Upon Wnt ligand binding, the activation complex forms at the membrane where the kinase activity of CK1 and GSK3 is redirected toward LRP5/6 in complex with Axin and Dsh. β-catenin is no longer phosphorylated, enters the nucleus, and takes part in transcription along with TCF; (**C**) The non-canonical Wnt pathway uses a variety of transmembrane proteins like Ror to affect cellular polarity both within the plane of the tissue (PCP) or even within the cell (apico-basal polarity). Figure was adapted from Schlesinger et al. [27].

Members of the Wnt family of secreted glycoproteins play evolutionarily conserved roles in establishing embryonic axes and in lineage specification during gastrulation [8]. Significantly, Wnts are also iteratively used beyond development and function prominently in adult tissue homeostasis. For example, a class of Wnts commonly referred to as “canonical” require β-catenin for their intracellular signal transduction, and these ligands play well-established roles in the maintenance of stem cell niches [10]. Of clinical significance, Wnt pathway dysregulation in these niches is intimately associated with tumorigenesis. Probably the most famous example is the hereditary colon cancer known as Familial Adenomatous Polyposis (FAP), which results from germline mutations in the *Adenomatous Polyposis Coli* (*APC*) gene [12]. In the absence of Wnt stimulation, APC (and other proteins: Axin, GSK-3, Figure 1) form a destruction complex that normally targets β-catenin for ubiquitylation. In FAP patients, loss of APC results in constitutive activation of the Wnt/β-catenin pathway and adenoma formation [13,14]. Generally, the mechanism of canonical Wnt signal transduction is based on phosphorylation and ubiquitin-mediated degradation of β-catenin, ensuring low levels of cytoplasmic of β-catenin and preventing its entry into the nucleus (Figure 1A). Upon Wnt ligand binding, an activation complex forms at the membrane preventing β-catenin degradation, allowing its protein levels to increase and translocation into the nucleus where it functions as a transactivator along with the transcription factor TCF (Figure 1B).

A second class of Wnts, aptly called “non-canonical”, engage diverse and less well characterized signal transduction pathways that do not employ β-catenin [15,16,17]. In a number of model systems, non-canonical Wnts have been shown to control epithelial apico-basal cell polarity (asymmetry within individual cells), cell organization within the plane of a tissue (so-called planar cell polarity or PCP), and cell migration during morphogenetic processes [18,19]. In contrast to canonical Wnts, they are unable to transform cells when overexpressed, and consequently their role in cancer was not immediately apparent. Wnt ligands can work as directional cues setting up asymmetry within a tissue or a cell [20,21]. Very recently, however, Wnt/PCP signaling pathway components—ligands, receptors, co-receptors and intracellular effectors—have been linked to tumor invasion and metastasis [22,23,24]. One recent example of this was the finding that autocrine Wnt-PCP signaling stimulated the motility and protrusion of breast cancer cells through endosomes loaded with Wnt11, leading to metastasis [25].

The best characterized, although not without its controversies, is the planar PCP pathway that organizes wing hairs and ommatidial orientation in *Drosophila* [15]. The vertebrate equivalents are also well studied, but fall into several pathways that may not be independent of each other [26,27]. Such is the variety and interplay between these pathways that a recent opinion piece, echoing a much earlier paper [28], suggested that non-canonical signaling could be viewed as a network of differing, cell-specific effects rather than as distinct linear pathways [29]. The PCP pathway mechanism is complex and described extensively elsewhere [19,26,30], but the very broad mechanism for Wnt-polarity pathways includes the cytoplasmic protein Disheveled indirectly activating small GTPases leading to cytoskeletal and adhesive changes in cells (Figure 1C, [27]). This review focuses on two proteins that do not fit the standard non-canonical signaling pathways: Protein tyrosine kinase 7 (Ptk7) and the multiple PDZ domain protein, Mutated in colorectal cancer (Mcc).

## 2. The Transmembrane Receptor Ptk7

Ptk7 is a single-pass transmembrane Wnt/PCP co-receptor known to control cell migration and polarity in the fly, chicken, frog, zebrafish and mouse embryos [31], and recently was found to affect regeneration in planarians [32]. In *Drosophila*, the *Ptk7* orthologue *off-track* (*otk*) was discovered as a neuronal pathfinding molecule. Otk associates with Plexin A to transduce a repulsive signal from Semaphorin 1a. Loss of Otk protein leads to embryonic axon guidance defects in the central nervous system and in motor neuron projections [33], as well as inappropriate photoreceptor cell connectivity in the fly eye [34,35].

The human Otk orthologue was originally named *Colon Carcinoma Kinase 4* (*CCK4*), a gene highly upregulated in colon cancers, but was renamed *Protein Tyrosine Kinase 7 (Ptk7)* because its protein structure places it among a single-pass transmembrane receptor family with a deficient kinase domain [36,37]. Early studies examined *Ptk7* expression in a variety of cancers, and revealed that *Ptk7* levels are higher in lung, colon and gastric cancers and are associated with poor prognosis and higher metastatic potential [38,39,40,41,42,43]. In contrast, *Ptk7* is downregulated in some subtypes of ovarian cancers and melanomas [44,45], but not other ovarian cancer subtypes [46]. Ptk7 has additionally been linked to breast cancer [47,48,49] and leukemia [50,51,52,53]. Recently, Ptk7 was implicated in cancer cell motility and metastasis in fibrosarcoma HT1080 cells, identified as a potential diagnostic biomarker for a variety of cancer types [54,55,56,57,58,59,60,61,62], and proposed as a tumor suppressor gene by inhibiting ERK and AKT phosphorylation in lung cancer [39].

An embryonic role for Ptk7 was first discovered through a gene trap screen for mouse mutations affecting neural development [63], and has subsequently been linked to human neural tube defects [64]. Loss of *Ptk7* resulted in neural tube closure and cochlear cell rotation defects, both considered classic mouse PCP phenotypes [30,65]. In *Xenopus*, targeting *Ptk7* transcripts with antisense morpholino oligonucleotides resulted in convergence and extension defects during gastrulation [63]. Convergence and extension is a morphogenetic process consisting of a specialized set of mass cellular rearrangements that simultaneously narrows the body axis mediolaterally and elongates it from head to tail. Subsequently, it was shown that mouse and zebrafish embryos also require Ptk7 for proper convergence and extension movements [66,67]. The *Ptk7* gene has been implicated in idiopathic scoliosis in a zebrafish model, where it appears to be required for directional cerebrospinal fluid flow regulated by ciliated cells [68,69]. In addition, much like the invertebrate function of Otk, in vertebrate development, Ptk7 interacts with plexin A1 regulating neural crest migration [70,71,72]. Ptk7 is cleaved by a membrane type matrix metalloprotease (MT1-MMP) affecting its function in both zebrafish and human development, and cancer cell metastasis [61,73,74,75,76]. Taken together, these findings suggest that Ptk7 is a highly regulated, polarity-determining molecule in a variety of cellular behaviors both during development and in cancers.

Recent work also revealed a strong link between Ptk7 and a variety of stem cell functions. For example, antibodies to the extracellular domain of Ptk7 can be used to isolate human colonic stem cells directly from patient samples, and importantly these cells are similar to LGR5+ gut stem cells [42]. Another study showed that mice lacking *Ptk7* had decreased pools of hematopoietic stem cells [77]. Taken together, these findings emphasize that Ptk7 plays critical roles not only during embryonic development but also in the maintenance of tissue homeostasis and cancer. Thus, one pressing experimental need is to identify the signals operating upstream of the Ptk7 transmembrane receptor and to determine how Ptk7 relays these extracellular signals intracellularly to coordinate such diverse processes [8,10,78].

## 3. Ptk7 and Wnt Signaling

The *Wnt* oncogene was originally identified as an integration site for mouse mammary tumor virus more than thirty years ago [9,79,80]. Upregulation of *Int-1* as it was known then caused mammary tumors, launching a field of basic research that defined the canonical Wnt pathway. More recently, several Wnt signaling pathways that function to generate different cellular outcomes have been discovered [2,3,4]. Wnt pathways affect polarity, stem cell maintenance and asymmetric cell division, cell growth and proliferation, differentiation, and apoptosis [3,27,78]. Non-canonical Wnts have been scrutinized for their role in cancer progression and metastasis (reviewed in [11,17,81]). Since all of these processes affect human health, much work has focused on differentiating one type of signaling from another, leading to a major biological question of how Wnt proteins can elicit such different outcomes in cells? There is great interest in this topic, as new Wnt pathway based therapies are being developed targeting specific cancers [82], but these could have inadvertent side effects by affecting other crucial systems such as stem cell maintenance.

**Figure 2:**
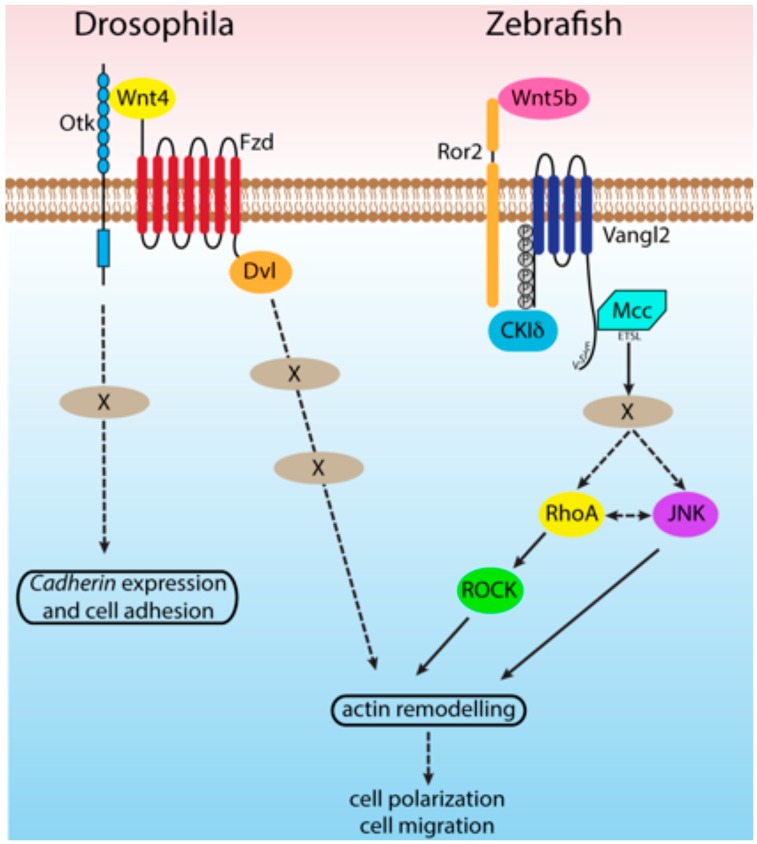
Comparison of the *Drosophila* and vertebrate non-canonical Wnt pathways applicable to this proposal. The Ptk7/Otk non-canonical Wnt pathway affects several polarity-related signaling molecules, but how direct these effects are is not known. These pathways function through effects on cytoskeleton, adhesion, and subcellular organization.

The discovery of Wnt co-receptors allowed the assorted non-canonical Wnt signaling pathways to be teased apart. These co-receptors show different affinities for different Wnt ligands, and elicit distinct cellular outcomes [83,84,85,86]. For example, the Wnt5-Derailed (Ryk in vertebrates) system regulates *Drosophila* embryonic axon guidance, whereas Egl20 (Wnt)-Ror in *C. elegans* organizes cells during oogenesis. Both examples show how discrete Wnt-co-receptors, either Derailed/Ryk or Ror, activate non-canonical signaling pathways [87,88]. The model then claims that the specific co-receptor recruited upon Wnt ligand binding activates a specific cellular pathway by engaging different intracellular signaling molecules [89,90]. This model requires a number of different co-receptors with different affinities for different Wnt ligands. Otk serves such a role as it specifically binds to *Drosophila* Wnt4 [91] and Wnt2 [92]. *Xenopus* Ptk7 selectively interacts with Wnt3a and Wnt8 [91]. This interaction requires Frizzled 7 (Fzd7), suggesting that Fzd7 is a Ptk7 co-receptor. Both Ptk7 and Otk oppose canonical β-catenin-dependent signaling during *Drosophila* and *Xenopus* embryonic development [91,93]. Mutations in *Ptk7* have been implicated in mouse neural tube closure defects [63], and loss of function mutations in *Zebrafish* show defects in axial convergence and extension, and neural tube morphogenesis [67]. Ptk7 also binds to the non-canonical ligand Wnt5a leading to Jnk activation and morphogenetic cell rearrangements [94]. *Wnt5a* and *Ptk7* mutant mice show very similar intestinal phenotypes, suggesting a mechanism where the non-canonical Wnt5a signals through Ptk7-expressing intestinal epithelial cells (Figure 2 [95]).

The specific role of Ptk7 in Wnt signaling remains controversial. Ptk7 recruits the Wnt intracellular effector Disheveled to the membrane, which is a crucial step in Dsh activation (Dsh in *Drosophila* and Dvl in Vertebrates) [91,96,97]. Dsh functions as a critical node in deciding which downstream pathway will be activated, and it appears to be the only Wnt pathway component that is involved in both canonical and non-canonical signaling. In several studies, this interaction leads to non-canonical signaling [94,96], but it can also activate canonical signaling, as a yeast two-hybrid interaction between β-catenin and Ptk7 was previously observed [98]. It remains unclear how the interaction between β-catenin and Ptk7 at the membrane activates canonical Wnt signaling, but Ptk7 is clearly required for the formation of the *Xenopus* Spemann organizer, which has been extensively characterized as reliant on canonical Wnt signaling [98]. In *Drosophila*, expression of Otk along with Wnt4 inactivates canonical signaling [91]. More controversially, a recent study using newly engineered deletions of *otk1* and *otk2* in *Drosophila* found a lack of any embryonic phenotypes casting some doubt on both neuronal pathfinding and Wnt signaling roles for Otk [92]. Clearly, further research will be required to describe the role or roles of Ptk7 in Wnt signaling, but at least in vertebrate systems there is clear evidence for Ptk7 functioning in the network of Wnt signaling pathways [31,72].

## 4. Role of Mcc

The gene *Mutated in Colorectal Cancer* was identified more than 25 years ago through linkage studies and positional cloning as a culprit tumor suppressor gene for colon cancer, as its name implies. Shortly after this initial finding, a now much more famous gene, *Adenomatous Polyposis Coli* (APC), which is tightly linked to *Mcc* on human chromosome 5q, was established as the gene responsible for hereditary colon cancer (FAP). As APC grew in prominence, with its binding to β-catenin first established in 1993 [99], interest in Mcc waned considerably. Several studies have appeared intermittently over the last two decades that have interrogated Mcc function biochemically in various cancer lines, and a few others have slowly emerged supporting a role for Mcc as a *bona fide* tumor suppressor in some cancers, including colorectal cancer, B cell lymphoma and hepatocellular carcinoma [100,101,102,103]. Significantly, Mcc, like its genetic neighbor APC, has also been implicated in WNT signaling, both canonical and non-canonical [104].

*Mcc* encodes an evolutionarily conserved, multiple PDZ (PSD-95/DLG/ZO-1) domain-containing protein. When overexpressed, Mcc binds β-catenin in the nucleus to negatively regulate canonical Wnt signaling in cancer cell lines and to inhibit cell proliferation [105,106]. Recently, a yeast two-hybrid screen identified Deleted in Breast Cancer 1 (DBC1) (officially Cell Cycle and Apoptosis Regulator protein 2 (CCAR2)) as an Mcc-interacting protein [107]. Mounting evidence suggests that DBC1 positively regulates β-catenin activity through the deacetylase SIRT1 [108,109]. Pangon et al. (2015) demonstrate that Mcc overexpression results in the re-localization of DBC1 to the cytoplasm and β-catenin deacetylation. Irrespective of the molecular mechanism, these data support a model whereby Mcc normally antagonizes canonical, β-catenin dependent Wnt signaling [107].

A surprising role for Mcc in the non-canonical branch of Wnt signaling emerged from the striking phenotypic similarities between zebrafish embryos depleted of *mcc* transcripts with antisense morpholinos and previously characterized zebrafish mutants in genes encoding known components of the non-canonical Wnt pathway, including *wnt5a/pipetail* and *vangl2/trilobite/strabismus* [104]. Both *mcc* morphants and these classic mutants show reductions in anterior development, a foreshortened and ventrally curved body axis and tightly packed somites. Such defects originate from impaired convergence and extension movements during gastrulation—the failure of cells to elongate, to orient mediolaterally and to intercalate to lengthen the body axis—and commonly result from the manipulation of β-catenin independent, non-canonical Wnt signaling. In mice, extensive genetic interactions were previously reported among *Wnt5a*, *Ror2* and *Vangl2* null alleles during diverse mammalian CE processes, such as neural tube closure, inner ear hair cell polarity and limb elongation [26], and we reasoned that as a principally cytoplasmic protein Mcc might serve as an intracellular effector of the evolutionarily conserved Wnt5a/Ror2/Vangl2 signaling axis during zebrafish CE. Like the PDZ domain-containing protein Disheveled mentioned previously, Mcc indeed physically associates with the Vangl2 cytoplasmic tail, and in epistasis experiments *mcc* overexpression can rescue the loss of *wnt5a*, *ror2* or *vangl2*. How Mcc conveys extracellular Wnt5a signals to the actin cytoskeleton remains unclear, but evidence from zebrafish implicates both Rho and c-Jun N-terminal kinase (Jnk) [104].

## 5. Connections between Ptk7 and Mcc

As mentioned previously, Ptk7 in some signaling contexts engages Fzd7 as a potential co-receptor. Biochemical studies have shown that Vangl2 and Fzd4 form a receptor complex that strongly activates Jnk in vitro [110], and in zebrafish knockdown of *fzd7a/b* results in CE phenotypes largely overlapping with loss of *mcc*, *wnt5a*, *ror2, scrib1, vangl2* as well as *ptk7* [67,104,111,112]. Whether multimeric cell surface receptor complexes comprising of Ptk7, Fzd and Vangl2 exist is an outstanding question. Moreover, Ptk7 was recently shown to physically interact with Ror2 in vitro, transducing extracellular Wnt5a signals via intracellular Jnk, and not surprisingly Ptk7 and Ror2 cooperate during convergence and extension in *Xenopus* embryos [94]. *Mcc* is expressed in the crypts of the adult mouse intestine and recent evidence in both mice and man supports a tumor suppressor role for Mcc in colorectal cancer [102,113]. Similarly, *Ptk7* is expressed in both the developing gut as well as in the adult intestinal epithelium [114], and there is mounting evidence that Ptk7 dysregulation is associated with the pathogenesis of human gastric and colorectal cancer [115]. Ptk7 is known to interact with PDZ domain containing proteins like Scrib1 in developmental processes such as inner ear PCP [30] and neural tube closure in mice [116], and has been additionally implicated in human neural tube closure defects [117]. Scrib1, much like Mcc contains PDZ domains and is associated with a variety of human cancers [112], suggesting that both could be part of the Wnt non-canonical signaling network. Taken together, these intersecting findings emphasize the complexity of the cell surface permutations available to non-canonical WNT ligands to dictate specific downstream cellular behaviors.

## 6. Conclusions

There are remarkably few signaling pathways that specify the cell types, organs, spatial organization and cellular behavior, during development of complex organisms. Wnt signaling suggests one answer to this conundrum, as it is not one pathway but an amalgamation of many. The study of these sub-pathways through analysis of our example of Ptk7 and Mcc will yield insights into control of cell migration and polarity in the fly, zebrafish and mouse embryo, especially if the various model organism research can be combined to bridge the large evolutionary distances. At present, vanishingly little is known about how extracellular Wnt/PCP ligands lead to the activation of either Ptk7 or Mcc and to remodeling of the actin cytoskeleton, but new experimental approaches such as CRISPR mutagenesis in vertebrates [68,118,119] and the study of morphogenesis in the late *Drosophila* embryo where PCP, Wnt, and apico-basal polarity come together should lead to new insights [120,121,122,123,124,125,126,127,128]. Overall, a better understanding of the signaling pathways that mediate cell behavior will not only lead to improved disease treatment, but will enhance our knowledge of how developmental disorders work. In cancer biology, understanding how co-receptors regulate which Wnt outcome results should lead to more targeted therapies that affect only cell proliferation and not homeostatic functions.
